# A cross-sectional analysis of green space prevalence and mental wellbeing in England

**DOI:** 10.1186/s12889-017-4401-x

**Published:** 2017-05-17

**Authors:** Victoria Houlden, Scott Weich, Stephen Jarvis

**Affiliations:** 1 Warwick Institute for Science of Cities, Senate House, University of Warwick, Coventry, CV4 7AL UK; 2 University of Sheffield, Sheffield, UK; 3 University of Warwick, Coventry, UK

**Keywords:** Health, green space, Mental wellbeing, Rural, SWEMWBS, Urban

## Abstract

**Background:**

With urbanisation increasing, it is important to understand how to design changing environments to promote mental wellbeing. Evidence suggests that local-area proportions of green space may be associated with happiness and life satisfaction; however, the available evidence on such associations with more broadly defined mental wellbeing in still very scarce. This study aimed to establish whether the amount of neighbourhood green space was associated with mental wellbeing.

**Methods:**

Data were drawn from *Understanding Society*, a national survey of 30,900 individuals across 11,096 Census Lower-Layer Super Output Areas (LSOAs) in England, over the period 2009–2010. Measures included the multi-dimensional Warwick-Edinburgh Mental Well-Being Scale (SWEMWBS) and LSOA proportion of green space, which was derived from the General Land Use Database (GLUD), and were analysed using linear regression, while controlling for individual, household and area-level factors.

**Results:**

Those living in areas with greater proportions of green space had significantly higher mental wellbeing scores in unadjusted analyses (an expected increase of 0.17 points (*95% CI* 0.11, 0.23) in the SWEMWBS score for a standard deviation increase of green space). However, after adjustment for confounding by respondent sociodemographic characteristics and urban/rural location, the association was attenuated to the null (regression coefficient *B* = − 0.01, *95% CI* -0.08, 0.05, *p* = 0.712).

**Conclusions:**

While the green space in an individual’s local area has been shown through other research to be related to aspects of mental health such as happiness and life satisfaction, the association with multidimensional mental wellbeing is much less clear from our results. While we did not find a statistically significant association between the amount of green space in residents’ local areas and mental wellbeing, further research is needed to understand whether other features of green space, such as accessibility, aesthetics or use, are important for mental wellbeing.

## Background

Mass migration and population growth over the last century have led to more than half of the world’s population residing in cities, creating a challenge for urban planners to efficiently accommodate new residents in a health promoting environment [1–3]. It has been suggested that mental health may differ between urban and rural areas, with studies contrasting in the direction of their conclusions [4–6]. Positive mental health and wellbeing have been linked to increased longevity, productivity and societal prosperity, but have also grown in prominence both politically and economically [7–12]. For example, the EU-level *Beyond GDP* (Gross Domestic Product) initiative was developed to be more inclusive of such social and environmental aspects of progress, by quantifying climate change, poverty and mental wellbeing, as well as the economy [13]. In the UK, results from the 2015 Annual Population Survey showed that, while mental wellbeing had on average increased over recent years, the divide between those rating their personal wellbeing at the highest and lowest levels had also grown, indicating a wellbeing inequality which needs to be addressed [14].

Mental wellbeing comprises two main components: the hedonic dimension, which includes happiness, life satisfaction and pain avoidance; and the eudaimonic dimension, which focuses on self-realisation, purpose in life and psychological function [15, 16]. Rather than just the absence of mental illness, mental wellbeing therefore encompasses aspects of positive affect, relaxation, functioning, personal relationships, life satisfaction and general happiness [17–19].

Emerging evidence suggests that aspects of the physical environment, and exposure to nature in particular, are often associated with higher levels of happiness and life satisfaction [20–22]. While these are important aspects of mental wellbeing, the relationship between green space and this multi-dimensional view of mental wellbeing remains relatively unexplored [20–24].

In urban environments, green space is considered to be any area of grass, trees or other vegetation, which in towns and cities is deliberately reserved for recreational, aesthetic or environmental purposes; this term therefore covers a range of green urban features, including parks, sports pitches and streetscape greenery. While abundant in rural areas, green spaces are usually designed into urban landscapes, typically at the expense of buildings. To encourage this to happen, the UK government sets out green space recommendations to encourage Councils to build these into each neighbourhood; these recommendations have been developed from government survey-based research and consideration of accepted walking distances between homes and green spaces [25].

Studies have sought to understand why green spaces seem to be beneficial for health and wellbeing. The theory of biophilia suggests that people pursue connections to nature; humans evolved in a natural landscape, where green spaces would have offered shelter, potential sources of food, and hence survival, so we may still experience positive feelings in such environments [26, 27]. Exposure to nature might enhance wellbeing by providing mental escape and restoration from fatigue, which is the focus for two key theories. Attention Restoration Theory proposes that effortful, directed attention is required to undertake everyday tasks, while the involuntary fascination which nature attracts provides an opportunity to rest the brain and regain concentration [28–30]. By contrast, it is suggested that urban environments may be less restorative, because of excessive stimuli and a need for directed attention to process these high levels of information [29, 31]. An alternative, the Stress Recovery Theory, argues that views of nature are the most beneficial for restoration, by helping stressed individuals recover a relaxed emotional state [32, 33]; these theories have been validated by a number of studies [31, 34–40]. It perhaps follows that individuals are often attracted to scenic environments, in particular trees, vegetation and water [1, 32, 41, 42], and so exposure to such landscapes may be valuable for happiness [22, 33, 43–45]. As well as these restorative mechanisms, it is theorised that green spaces may contribute to better health by enabling activities known to promote mental wellbeing, such as social interaction [2, 19, 46, 47] and physical activity [21, 48].

Recent research has begun investigating the association between the proportion of green space in neighbourhoods and residents’ mental health and wellbeing [8, 21, 23, 49, 50]. One study found a positive association to a single life satisfaction measure, by analysing 10,000 individuals living in Lower-Layer Super Output Areas (LSOAs) in urban England [22]. Other work has demonstrated that socioeconomic inequalities in mental wellbeing (indexed by the WHO-5 positive wellbeing index) tend to be smaller among those who feel they have good access to recreational areas within their urban neighbourhood, although this study did not objectively quantify green space, or restrict recreational areas to those that were specifically green [8]. Several studies also report that people are more likely to have lower levels of mental distress, as measured by the General Health Questionnaire (a psychiatric screening tool), when residing in areas with relatively more green space [22, 23, 51]. One such longitudinal study reported that ward-level proportions of green space were negatively associated with psychiatric morbidity, although the strength of this association varied across life course and by gender [52]. While lower levels of psychiatric symptoms are generally associated with better wellbeing, as described, mental wellbeing is a positive measure which reflects much more than an absence of distress [53].

While studies in this area tend to examine aspects of positive mental health, such as relaxation, satisfaction and general happiness [1, 22, 32, 41–45, 54, 55], we are only aware of one other study implementing a multi-dimensional measure of mental wellbeing. The study was based on a small selective sample in deprived areas of Scotland, and investigated the association between local green space proportions and mental wellbeing, of which the results were mixed and inconclusive [56].

Previous studies have tended to consider either urban green space or the wider benefits of contact with nature; while urban-rural differences in health have been studied, it is not yet known whether the association between green space and mental wellbeing in particular differs in urban and rural areas [1, 31, 32, 57, 58]. Although urbanisation reduces opportunities for people to interact with natural environments, it remains unclear whether or how this might affect the mental wellbeing of those who live in cities [59, 60].

The primary aim of this research was to test two hypotheses: (1) that neighbourhood areas of England with greater proportions of local-area green space are associated with higher levels of mental wellbeing; and (2) that the association between the proportion of local area green space and mental wellbeing may be confounded and/or modified by urban versus rural location.

## Methods

### Design

#### Sample

Data were drawn from the first wave of the UK Longitudinal Household Panel Study (UKLHS), known as *Understanding Society*, which ran from 2009 to 2010 [61]. Only residents of England were included, because of the availability of land use data. The UKLHS is a biennial survey of people aged 16 and over in a sample of private households across England, Scotland, Wales and Northern Ireland. Households were selected via random sampling of individual addresses within specific postcode sectors, to optimise sampling efficiency [62]. The wave 1 sample contained 50,994 individuals, from 30,169 households. Each household is also given a local-area identifier, by special licence access, which can be used to link UKLHS to the geographical green space data. These Lower-Layer Super Output Areas (LSOAs) are standardised UK Census units ideal for examining spatial data. England is divided up into 32,844 LSOAs, each of which contains 400–1200 residences and, within this data set, covers an average area of 4.2km^2^ (sd 12.8km^2^).

#### Mental wellbeing

Mental wellbeing was measured using the Short Warwick-Edinburgh Mental Well-Being Scale (SWEMWBS), which is comprised of 7 positively-worded questions relating to both hedonic and eudaimonic aspects of positive mental health [18, 61]. The questionnaire, issued through the *Understanding Society* survey, asked respondents to rate how they have been feeling “over the last 2 weeks” on 7 domains: optimistic about the future, useful, relaxed, close to other people, dealing with problems well, thinking clearly, and able to make up one’s mind. Using a 5-point Likert scale, options are “none of the time” (score 1), “rarely”, “some of the time”, “often” and “all of the time” (score 5). This results in a final rating between 7 and 35, with a higher number indicating better mental wellbeing [18].

#### Individual and household-level confounders

Potential confounders of the association between green space and mental wellbeing were identified from the literature, as well as examination of the individual data available within *Understanding Society* [21–23, 49, 51, 56, 63]. These included ten-year age group, gender, marital status (single/unmarried, married/civil partnership, and separated/divorced/widowed), ethnicity (white British, white other, black, South Asian, other), and total number of serious on-going physical health conditions (continuous, including clinical diagnoses of, for example, epilepsy, heart disease, cancer). Socioeconomic status was assessed by means of employment status (unemployed, employed and economically inactive), household income (quintiles adjusted for household composition [64]), household space (bedrooms per person, categorised into <1, 1–3, > 3), living alone, living with children, and housing tenure. Data on commuting time to work was also included, in line with previous work [22, 23, 65]. Local-area deprivation, at the LSOA level, was controlled for using the English Index of Multiple Deprivation (IMD), which provides a score based on aspects including local education, income and crime statistics [66].

#### Green space

Green space data were obtained from the 2005 General Land Use Database (GLUD) [67], which provides land cover information for each LSOA in England. Each LSOA is given a total land cover and then divided into 9 usage categories, derived from Ordnance Survey’s MasterMap using visual inspection and information from the land registry; these groupings are domestic buildings, non-domestic buildings, domestic gardens, green space, water, path, road, rail, and ‘other’ [67]. For the purposes of this research, domestic gardens were not included as green space, as the category provided in the dataset included all domestic outdoor space, and so it could not be guaranteed that this was green. The relative amount of green space for each locality was calculated by dividing the area of green space by the total area for each LSOA, giving a proportion between 0 and 1.

#### Rural-Urban classification

Also included within the *Understanding Society* data [61], this Rural-Urban Classification divides England’s LSOAs into categories according to their level of urbanicity, based on population [68]. At the broadest level, urban centres are defined as settlements with a residential population greater than 10,000; as such, any local area is classified as urban if over 74% if its resident population lives in such an urban settlement. Within this dataset, the number of residents in urban areas, *n*, total 25,547; the rest are considered rural (*n* = 5353). This widest classification was selected for broad comparison and to ensure adequate amounts of data within each group.

### Analysis

Analysis began by describing the distributions of mental wellbeing and green space, along with the characteristics of the study sample. To test for potential confounding, and to avoid collinearity, associations were estimated between each individual variable and green space and mental wellbeing, in turn. Those that were associated with both variables to a statistically significant degree met the selection criteria and were therefore considered to be potential confounders. Included in the final dataset were: sex, age, marital status, ethnicity, health conditions, employment, income, household space, living alone, living with children, housing tenure and commuting time to work.

As exploratory analyses revealed the distribution of SWEMWBS to be moderately skewed, we investigated the variance of this output in order to determine the most appropriate modelling technique. Linear regression modelling, found to be the most suitable, was used to estimate the association between mental wellbeing (SWEMWBS score) and the proportion of green space in each LSOA. Survey commands in the R Survey package were used to control for the clustered sampling of participants within the primary sampling units (PSUs); these are a stratified sample of postcodes designed to be representative of the UK population, in both economic and ethnic terms. The use of survey commands in R allowed us to generate robust estimates of variance in the association between individual exposure to green space and mental wellbeing that took account of autocorrelation (and therefore higher-level variances) in the dataset.

In the unadjusted model, using SWEMWBS score as the dependent variable, the regression coefficient (*B*) for green space represents an estimate of the amount by which wellbeing score increases a standard deviation increase in green space. To adjust for potential confounders, multivariate models were then built, including individual, socio-economic, place and household variables. The adjusted regression model was then run using urban/rural location as an additional variable. Analyses were completed with R 3.1.2 [69] using the Survey package [70], and Stata [71].

## Results

In total, 50,994 individuals were included in wave 1 of the study, from 30,169 different households, which equates to a 57.6% participation response from the initially selected households, followed by an 81.8% individual-level response rate to the questionnaires issued to these agreeing households [72]. Little direct information was available regarding the characteristics of non-responding individuals, although they may be compared in terms of local-area socioeconomic statistics. The data collectors (*Understanding Society*) observed slightly lower response rates in areas with higher proportions of single-person households (59.0% response in 1st quartile of single-person households, compared to 55.5% in the highest,4th, quartile) and people in full-time employment (59.7% response in 1st quartile, 56.6% in 4th). Similarly, at the individual level, response rates were somewhat higher in areas of lower deprivation, in terms of Council Tax band (86.2% response in the lowest band A, 79.5% response in the highest bands E-H), suggesting a modest association between socio-economic status and survey participation [72].

Of the responding individuals, 42,972 were residents of England. After removing those who had missing SWEMWBS (mental wellbeing) scores, the final sample contained 30,900 individuals, from 19,684 different households, which is 61.0% of the original sample from the UKLHS. The sample covers 11,096 LSOAs across England, which vary considerably in size between urban (mean 0.9km^2^, sd 2.3km^2^) and rural areas (mean 19.6km^2^, sd 25.1km^2^). Of those not completing the mental wellbeing questions, mean green space exposure was 0.36 (sd 0.28), which was lower than the final sample (mean 0.42, sd 0.30) (Significance of t-test, *p* < 0.001).

From a socioeconomic perspective, local-area deprivation was significantly greater among SWEMWBS non-completers (mean score 27.1, sd 17.2 versus, 22.2, sd 15.6)(*p* < 0.001), although average equivalised income was consistent (£5515/month, sd £5438 for responders versus £5511/month, sd £5970 for non-responders) (*p =* 0.831).

In the final sample, prevalence of local area green space, given as a proportion of each LSOA, had a mean value of 0.42 (sd 0.30), with values of 0.33 (sd 0.24) and 0.82 (sd 0.19) in urban and rural areas, respectively. SWEMWBS scores were slightly negatively skewed; the mean score for the sample as a whole was 25.2 (sd 4.5), with a modal value of 28.0, and was significantly lower in urban than rural areas (mean score 25.1 (sd 4.6) versus 25.6 (sd 4.3))(*p* < 0.001).

The characteristics of people living in urban (*n* = 25, 547*)* and rural (*n =* 5353*)* areas also differed. The mean age of respondents was higher in rural areas, which also had greater proportions of married individuals. Income was also higher in rural areas, where area-level deprivation was considerably lower, household space was greater and more people owned their own home. These findings are presented in Table 1; t-tests were used to estimate the significance of the difference between urban are rural variables.

**Table 1:** Descriptive Statistics for the UK Longitudinal Household Survey, Data Sample

		All UKLHS Observations		Urban Only	Rural Only	*p for urban/rural differences*
*Variable*	*Value*	*n*	*mean (sd)/%*	*mean (sd)/%*	*mean (sd)/%*	
Individuals		30,900		25,547	5353	
Green space proportion		30,900	0.42 (0.30)	0.33 (0.24)	0.82(0.19)	<0.001
SWEMWBS		30,900	25.2(4.5)	25.1(4.6)	25.6(4.3)	<0.001
Sex	Male	13,679	44.3	45.8	44.0	0.701
	Female	17,221	55.7	54.2	56.0	0.701
Age	16–24	4421	14.3	15.2	10.0	<0.001
	25–34	5199	16.8	18.2	10.2	<0.001
	35–44	6145	17.5	20.4	17.3	<0.001
	45–54	5395	17.5	17.2	18.6	0.140
	55–64	4597	14.9	13.8	20.1	<0.001
	65+	5143	16.6	15.2	23.7	<0.001
Marital Status	Single	9800	31.7	33.8	21.8	<0.001
	Married	15,810	51.2	49.4	59.5	<0.001
	Post Marriage	5278	17.1	16.7	18.7	0.001
Ethnicity	White, British	23,997	77.7	73.8	96.1	<0.001
	White, Other	1151	3.7	4.0	2.5	<0.001
	Black	1863	6.0	7.2	0.2	<0.001
	South Asian	2670	8.6	10.4	0.4	<0.001
	Other	1193	3.9	4.5	0.7	<0.001
Health Conditions	Total number of clinically diagnosed serious conditions	30,900	0.5(0.9)	0.5(0.9)	0.6(0.9)	<0.001
Employment	Unemployed	1960	6.3	7.0	3.4	<0.001
	Employed	16,993	55.0	55.0	54.9	0.866
	Economically Inactive	11,947	38.7	38.0	41.6	<0.001
Income, Quintiles (mean)	1st	6180	£6385	18.6	13.5	<0.001
	2nd	6180	£11,241	19.8	17.6	<0.001
	3rd	6180	£15,085	20.4	20.2	0.693
	4th	6180	£20,059	20.9	22.0	0.550
	5th	6180	£36,127	20.3	26.6	<0.001
Household Space	<1 rooms per person	9622	31.1	33.2	21.3	<0.001
	1–3 rooms per person	20,917	67.7	65.8	76.6	<0.001
	>3 rooms per person	1749	5.7	5.4	7.1	<0.001
Living Alone		4504	14.6	14.8	13.7	0.032
Living with Children		10,822	35.0	36.4	28.5	<0.001
Housing Tenure	Own Home	20,849	67.5	65.6	76.4	<0.001
Commuting	<15mins	6392	20.7	20.9	19.8	0.064
	15–30 min	4760	15.4	15.7	14.2	0.004
	30–50 min	2107	6.8	6.9	6.3	0.065
	>50mins	1757	5.7	6.0	4.1	<0.001
IMD	Continuous	30,900	22.2(15.6)	24.1(16.2)	13.5(7.6)	<0.001

The unadjusted regression coefficient, *B*, for the association between proportion of green space and mental wellbeing was 0.17 points (*95% CI* 0.11, 0.23) in the SWEMWBS score, per standard deviation increase in green space. After controlling for all individual and household-level confounding factors (apart from urban/rural location), this coefficient was reduced 0.01 points (−0.05, 0.07) (*p* = 0.774).

Finally, adjusting further for urban/rural location in the association between a standard deviation increase in green space and SWEMWBS score, the resultant *B* value was −0.01 points (−0.08, 0.5, *p* = 0.712). While green space and urbanity were significantly linearly associated (*B* = −0.23, *p* < 0.001), we only found slight, but statistically insignificant evidence of effect modification (*B* = −0.11, *95% CI* -0.29, 0.11, *p* = 0.382) between these variables. Stratified univariate models showed that the association was slightly stronger in rural (*B* = 0.12 points, *95% CI* -0.01, 0.21, *p* = 0.062) than urban areas (*B* = 0.07 points, *95% CI* 0.01, 0.13, *p* = 0.027), for a standard deviation increase in green space, although only the urban result was statistically significant.

The results of the fully-adjusted model are presented in Table 2.

**Table 2:** Fully Adjusted Linear Regression Model

*Variable*	*Value*	*B (95% CI)*	*p*
Proportion of Green Space	(sd increase)	-0.01 (−0.08, 0.05)	0.712
Sex	*Male as reference*		
	Female	−0.07 (−0.16, 0.18)	0.164
Age	*16–24 as reference*		
	25–34	−0.34 (−0.56, −0.12)	0.002
	35–44	−0.86 (−1.09, −0.63)	<0.001
	45–54	−0.90 (−1.14, −0.66)	<0.001
	55–64	0.28 (0.02, 0.54)	0.032
	65+	1.24 (0.96, 1.52)	<0.001
Marital Status	*Married as reference*		
	Single/Unmarried	−0.69 (−0.86, −0.53)	<0.001
	Separated/Divorced/Widowed	−0.69 (−0.86, −0.52)	<0.001
Ethnicity	*White, British as reference*		
White, Other		0.42 (0.14, 0.69)	0.003
Black		1.01 (0.76, 1.26)	<0.001
South Asian		0.28 (0.05, 0.52)	0.019
Other		0.18 (−0.11, 0.47)	0.224
Health Conditions		−0.63 (−0.69, −0.57)	<0.001
Employment	*Employed as reference*		
	Unemployed	−1.10 (−1.35, −0.035)	<0.001
	Economically Inactive	−0.38 (−0.53, −0.23)	<0.001
Income, Quintiles	*1st as reference*		
	2nd	0.24 (0.06, 0.43)	0.010
	3rd	0.29 (0.10, 0.47)	0.002
	4th	0.67 (0.48, 0.86)	<0.001
	5th	0.94 (0.75, 1.13)	<0.001
Household Space	*1–3 rooms per person as reference*		
	<1 room per person	−0.08 (−0.22, 0.06)	0.258
	>3 rooms per person	0.19 (−0.09, 0.46)	0.18
Living Alone	*No as reference*		
	Yes	−0.06 (−0.27, 0.15)	0.576
Living with Children	*No as reference*		
	Yes	−0.18 (−0.32, −0.03)	0.018
Housing Tenure	*Does not own home as reference*		
	Own Home	0.32 (0.19, 0.46)	<0.001
Commuting Time	*<15 mins as reference*		
	15–30 min	0.03 (−0.11, 0.18)	0.664
	30–50 min	0.06 (−0.14, 0.26)	0.561
	>50 mins	0.27 (0.06, 0.49)	0.012
Deprivation		−0.02 (−0.02, −0.01)	<0.001
Urban/Rural Setting	*Rural as reference*		
	Urban	−0.10 (−0.27, 0.08)	0.283

As a sensitivity analysis, we repeated these models using quasi-poisson and log-transformed regressions, to account for the skewed distribution of the SWEMWBS variable. These modelling techniques did not significantly change our findings.

## Discussion

### Main findings

Previous research has demonstrated local-area prevalence of green space to be positively related to life satisfaction, happiness and reduced risk of psychiatric morbidity [22, 23, 51, 52]. In particular, studies applying data from the British Household Panel Survey (the predecessor to *Understanding* Society, which collected similar data), have shown a significant association between proportion of local area green space and lower GHQ scores, which held across longitudinal analyses [22, 23, 52]. We failed to find such an association when using a multi-dimensional measure of mental wellbeing as the study outcome, after adjusting for a wide range of potential confounders. These differences may be methodological, as we controlled for local-area deprivation and urban/rural location, as well as modelling green space as a continuous proportion, while Astell-Burt et al. did not [73]. However, White et al. found significant associations between green space and GHQ in their urban-area studies, while controlling for similar potential confounders, which, compared to our results, suggests that mental wellbeing reflects more than an absence of mental distress [22].

Although we hypothesised that urban/rural location may modify any associations between green space and mental wellbeing, we did not find any evidence supporting such an effect modification.

It may be useful to speculate on the processes underlying the observed confounding of the association between green space and mental wellbeing. For example, it has been suggested that levels of community and social support may be lower in rural areas, where people may be more isolated (perhaps because of difficulties accessing transport, or through fewer opportunities to socialise in remoter rural areas) [4]. Similarly, services (health and otherwise) may be less accessible in rural areas. However, we also note that our estimates were limited by the smaller sample of those living in rural areas, where variance in the proportion of green space was smaller than that observed in urban areas. Our findings should therefore be interpreted with caution.

These findings may also reflect methodological limitations, such as only including LSOA-level green space prevalence, or conceal more nuanced associations between green space and mental wellbeing. Green space itself may take many forms, and it may be that the association with mental wellbeing depends on the type rather amount of green space [74, 75]. Similarly, previous studies have shown that the quality of green space, and its biodiversity, were positively associated with mental health, where quantity was found to be less significant [76]. Context is also likely to matter [57, 58] and studies show that places that look untended or are poorly lit may be perceived as unsafe [47, 77, 78].

### Strengths and limitations

To the best of our knowledge this is the first study to test the association between green space and a multi-dimensional mental wellbeing measure that includes both eudaimonic and hedonic mental wellbeing items, in all parts of England. The UKLHS is the largest household survey in the UK to date [22, 23], and contains extremely detailed socio-economic data as well as spatial identifiers. The latter allowed us to link the survey data to land use data, and to compare the effects of urban/rural location on mental wellbeing and on the association between green space and mental wellbeing.

Despite the strengths of this work, the quantification of green space is relatively simplistic, and it is possible that associations with mental wellbeing were not detected as a result of grouping all types of green space into one variable.

It is also possible that the attribution of green space scores according to the value for LSOAs introduced an element of misclassification, since it takes no account of accessibility or interaction with this space. As the LSOAs are derived according to population, neighbourhoods in urban areas will naturally be much smaller geographically than those in sparser settings, thereby making adjacent areas in built-up environments more accessible to these residents. Future research which includes data on distances to the nearest green space (which may extend to that in adjacent LSOAs) might demonstrate larger associations with mental wellbeing. These data were limited to the green space in the LSOA of residence, and did not take account of where respondents worked or spent time, or areas traversed when commuting. At the individual level, there was evidence of greater response rates in less deprived areas, a possible source of selection bias. Finally, our cross-sectional study, by design, had a limited capacity to establish causality.

## Conclusions

The proportion of green space in an individual’s local area was significantly and positively associated with mental wellbeing in univariate models, but became weaker and statistically non-significant after adjusting for socio-demographic variables and urban/rural location. While the green space in an individual’s local area has been shown to be related to aspects of mental health such as happiness and life satisfaction, the association to multi-dimensional mental wellbeing is much less clear. Further research is therefore needed to explore the relationship of other aspects of green spaces aside from size, such as accessibility, aesthetics and use, to mental wellbeing.

## Abbreviations

GDP: Gross domestic product
GLUD: General land use database
LSOA: Lower layer super output area
SWEMWBS: Short warwick-edinburgh mental well-being scale
UKLHS: UK Longitudinal household panel study
